# Recent Advances in Microglia Modelling to Address Translational Outcomes in Neurodegenerative Diseases

**DOI:** 10.3390/cells11101662

**Published:** 2022-05-17

**Authors:** Carla Cuní-López, Romal Stewart, Hazel Quek, Anthony R. White

**Affiliations:** 1 Cell & Molecular Biology Department, Mental Health Program, QIMR Berghofer Medical Research Institute, Brisbane, QLD 4006, Australia; carla.cuni-lopez@qimrberghofer.edu.au (C.C.-L.); romal.stewart@qimrberghofer.edu.au (R.S.); 2 Faculty of Medicine, The University of Queensland, Brisbane, QLD 4006, Australia; 3 UQ Centre for Clinical Research, The University of Queensland, Royal Brisbane & Women’s Hospital, Brisbane, QLD 4006, Australia

**Keywords:** neuroinflammation, microglial platforms, patient-derived microglia cells, clinical translation, patient heterogeneity

## Abstract

Neurodegenerative diseases are deteriorating conditions of the nervous system that are rapidly increasing in the ageing population. Increasing evidence suggests that neuroinflammation, largely mediated by microglia, the resident immune cells of the brain, contributes to the onset and progression of neurodegenerative diseases. Hence, microglia are considered a major therapeutic target that could potentially yield effective disease-modifying treatments for neurodegenerative diseases. Despite the interest in studying microglia as drug targets, the availability of cost-effective, flexible, and patient-specific microglia cellular models is limited. Importantly, the current model systems do not accurately recapitulate important pathological features or disease processes, leading to the failure of many therapeutic drugs. Here, we review the key roles of microglia in neurodegenerative diseases and provide an update on the current microglial plaforms utilised in neurodegenerative diseases, with a focus on human microglia-like cells derived from peripheral blood mononuclear cells as well as human-induced pluripotent stem cells. The described microglial platforms can serve as tools for investigating disease biomarkers and improving the clinical translatability of the drug development process in neurodegenerative diseases.

## 1. Introduction

The global increase in incidence and public health burden of age-related neurodegenerative diseases requires effective prevention and treatment strategies [1]. Neurodegenerative diseases including Alzheimer’s disease (AD), Parkinson’s disease (PD) and amyotrophic lateral sclerosis (ALS) are symptomatically characterised by the impairment of cognitive and/or motor functions. Such symptoms result from neuronal cell death, which has been linked to the presence of toxic protein deposits in the central nervous system (CNS). Examples of proteins that misfold and form pathological aggregates associated with neurodegenerative diseases include amyloid-β (Aβ) and tau for AD; α-synuclein for PD; and superoxide dismutase 1 (SOD1) and TAR-DNA-binding protein (TDP-43) for ALS (*for an in-depth review on the toxic role of protein aggregation in neurodegenerative diseases*, *see* [2]).

Drug candidates targeted at reducing the accumulation of toxic protein deposits have repeatedly failed to ameliorate disease symptoms in patients [3]. Drug failure in neurodegenerative diseases is greater than 99% and is often evident in the later stages of clinical drug development [4]. Hence, improving the early phases of the drug development pipeline could significantly enhance drug outcomes for neurodegenerative diseases. Two approaches towards increasing the success of clinical trials include exploring alternative therapeutic targets and using relevant model systems of disease.

The limited clinical success of trialled therapeutic targets, such as toxic protein deposits, highlights the complex interplay of cellular mechanisms driving neuronal degeneration in neurodegenerative diseases. One such mechanism is unresolved neuroinflammation, which is primarily orchestrated by microglia, the specialised brain-resident macrophages. Microglia respond to toxic protein deposits by triggering neuroinflammatory responses that can lead to neuronal cell death and exacerbate disease if unresolved [5,6]. Microglia-mediated neuroinflammatory responses are highly dependent on disease stage, sex and patient genetic make-up [7]. Hence, microglial function may be linked to the clinical heterogeneity observed in neurodegenerative diseases. Such clinical heterogeneity could underlie the lack of success of ‘one-size-fits-all’ therapeutic approaches tested for neurodegenerative diseases. These interventions have translated poorly to patients despite showing promising outcomes in pre-clinical studies [8]. A reason for this poor translatability is the limited representation of patient-specific disease traits in the microglia models used. Therefore, using patient-specific disease models to investigate the therapeutic modulation of microglial activity could hold the key to more successful neurodegenerative disease drug development strategies at a personalised level.

## 2. The Role of Microglia in Neurodegenerative Diseases

Microglia are a type of glial cell with immune functions specific to the CNS. Their roles in the CNS span more than just immune sentinels. They provide trophic support to neurons, participate in neurogenesis, phagocytose cellular debris and dysfunctional synapses and remodel neural circuits (*for a review,* see [9]). With such a critical contribution to maintaining CNS homeostasis, it is not surprising that alterations to the microglial compartment have been implicated in a vast array of neurological diseases (*for a review,* see [10]).

In neurodegenerative diseases, the pathological build-up of misfolded proteins in the brain activates an immune state in microglial cells. Indeed, microglia are highly sensitive to their local microenvironment and can rapidly respond through a broad repertoire of activation states. These microglial states aim to protect the integrity of the CNS against threatening agents and activate downstream signalling pathways that increase microglial phagocytic clearance and the secretion of inflammation-related molecules (e.g., cytokines and chemokines) [11]. In the case of AD, pathways controlling these microglial states are regulated by genes that express high-risk variants. For example, *TREM2*, *CD33* and *PLCG2* regulate microglial immune functions and accumulate approximately 25% of AD risk variants [12]. These variants not only confer risk or protect against developing AD, but also strongly correlate with the severity of disease in patients [13].

In other neurodegenerative diseases such as PD and ALS, microglia exhibit alterations in molecular pathways that resemble those in AD. Specifically, key microglial functions such as sensing exogenous stimuli and danger signals and the release of inflammatory responses become defective [14,15]. Moreover, similar to AD, a change in microglial phenotypes correlates with disease progression in PD and ALS patients [16,17].

Taken together, microglia and their downstream signalling cascades offer promising options for targeted therapeutics as an alternative to or in combination with strategies to improve the clearance of protein deposits in neurodegenerative diseases.

## 3. Microglia as a Therapeutic Target for Neurodegenerative Diseases

Neuroinflammation, largely mediated by microglia, is a prominent hallmark in the brains of neurodegenerative disease patients [18]. It consists of increased levels of pro-inflammatory mediators, including tumour necrosis factor (TNF), interleukin (IL)-1β, IL-6, prostaglandins and reactive oxygen species (ROS), which persist at all disease stages [19]. Such molecules are detected in abnormal levels in patients’ cerebrospinal fluid and blood and can be used to predict disease progression from asymptomatic to more severe stages [20]. Early on in disease, neurons trigger a mild inflammatory activation of microglia to gain protection from toxic protein aggregates. Subsequently, the inflammatory state in microglia exacerbates and leads to a persistent release of harmful pro-inflammatory molecules that eventually causes irreversible neuronal damage.

Shifting microglia from harmful to beneficial phenotypes is a promising strategy for creating more effective disease-modifying drug candidates. Currently, 18% of disease-modifying agents in phase 3 clinical trials of AD have immunomodulatory actions targeting microglial biology [21,22]. In ALS, a phase 2 trial showed an improvement of disease progression in patients with masitinib, a selective tyrosine kinase inhibitor that blocked microglial proliferation [23]. Drug candidates targeting microglial biology are also in the pipeline for PD and are currently in the early stages of clinical testing [24]. Overall, these clinical trials indicate the current interest in targeting microglial cells as a therapeutic intervention in neurodegenerative diseases.

Microglia-modulating therapeutic candidates are likely to face challenges in efficacy trials for neurodegenerative diseases. These difficulties stem from the lack of a well-defined biological target and unclear biomarkers of suitable treatment time windows [25]. To address such limitations, disease model systems that capture microglial states during the course of the disease in patients may increase the success of candidate treatment strategies.

## 4. Obstacles to Modelling Microglia for Pre-Clinical Studies

To reliably screen microglia-targeted therapeutic candidates for neurodegenerative diseases, the use of accurate pre-clinical model systems is essential. An obstacle that hampers the accuracy of these models is their ability to capture specific features of the microglial identity, which is unique to humans and differs from the rest of specialised macrophages [26]. Another challenging aspect of experimental microglia models is the capacity to recapitulate the spectrum of phenotypes that these cells display in response to the changing microenvironmental cues, associated with the CNS region, ageing, sex and disease-related processes [27,28].

### 4.1. Species-Specific Microglia Signature

**Figure 1:**
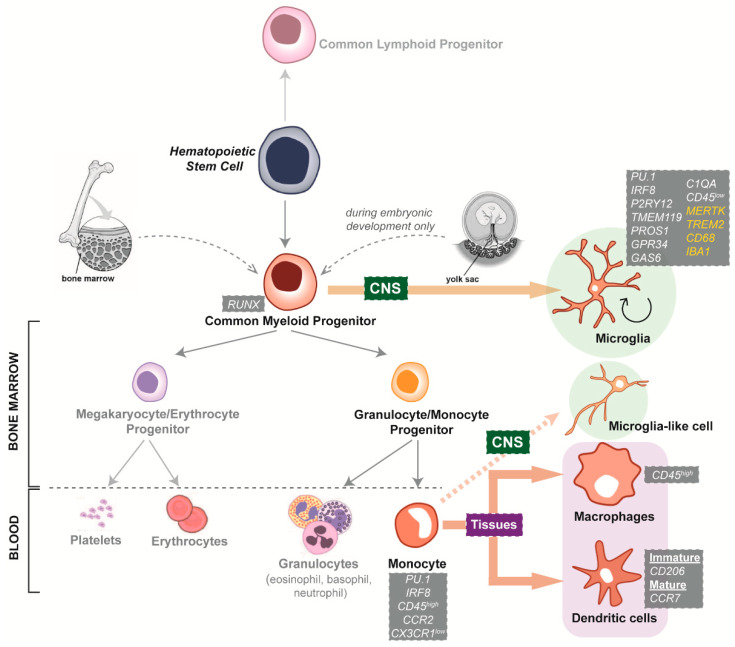
Schematic illustration of the development of myeloid cells. Hematopoietic stem cells can commit to either a lymphoid or a myeloid fate through the generation of common lymphoid or myeloid progenitor cells. Common myeloid progenitors are located in the bone marrow of adults, and the yolk sac in embryos. When yolk sac-derived myeloid progenitors colonise the CNS, specific microenvironmental cues direct their differentiation into microglia. These embryonically derived microglia are able to proliferate and self-maintain until adulthood. In the bone marrow, common myeloid progenitors differentiate towards megakaryocytic/erythrocytic or granulocytic/monocytic phenotypes. In the blood, megakaryocyte/erythrocyte progenitors give rise to platelets and erythrocytes (red blood cells), while granulocyte/monocyte progenitors give rise to leukocytes, including granulocytes and monocytes. Circulating monocytes can then be recruited to sites of infection or injury in specific tissues and differentiate into macrophages or dendritic cells. During aging and certain inflammatory conditions, monocytes and other bone marrow-derived progenitors infiltrate the CNS and differentiate into microglia-like cells. It is not well understood whether these microglia-like cells persist or are a temporary addition to the existing microglial population. Listed in grey boxes are representative markers expressed by myeloid cell types. Common markers between microglia and macrophages are highlighted in yellow (*MERTK, TREM2, CD68* and *IBA1*).

Microglia originate from primitive macrophages that migrate from the yolk sac, a mesoderm-derived extra-embryonic structure, into the developing CNS [26]. Exposure to environmental cues of the CNS during embryogenesis strongly shapes the identity of microglial cells, where the genetic and transcriptomic microglial signature is unique among tissue macrophages. For example, microglia-specific genes such as *TMEM119* and *P2RY12* are not expressed by other brain cells or myeloid cell types [29]. Conversely, other markers such as *C1QA, PROS1, GPR34* and *GAS6* show selective enrichment in microglia but are typically present in other myeloid cells (Figure 1).

The genetic signature of microglia also differs across species. This includes a subset of genes related to innate immune functions, which is expressed at very low levels in murine compared to human microglia [30,31]. In addition, pathways associated with the complement system, apoptotic cell clearance and metabolic activities such as ferroptosis are significantly enriched in microglia from humans compared to mice [32].

Microglial homeostatic genes regulate functions such as ramification and motility, synaptic remodelling, and immune vigilance, and are altered in the context of neurodegeneration [33,34]. Indeed, the dysfunction of such microglial roles significantly contributes to the neurodegenerative pathology and exhibits species differences. For example, homeostatic microglial genes are upregulated in AD patients but downregulated in mouse models [35]. In addition, only a fraction of AD and PD susceptibility genes expressed by human microglia are observed in rodent models [32]. These differences highlight the importance of using human cellular models to better investigate microglial alterations in the context of neurodegenerative diseases.

### 4.2. Microglial Dependence on the Surrounding CNS Microenvironment

The strong plastic capacity of microglia allows them to adjust their homeostatic profiles to a varied range of changing CNS environmental factors. These factors derive from ageing and/or diseased states, allowing microglia to exert tailored behaviours to restore homeostasis. Even under homeostatic conditions, a diversity of microglia subsets, each with specialised functions, co-exist within the CNS [28,36]. In the different CNS anatomical regions, variations in the surrounding microenvironments lead to microglia populations with different phagocytic, activation and proliferation capacities [37,38,39]. Moreover, these CNS region-specific microglia populations exhibit unique morphological features, lysosome contents, membrane properties and transcriptomes [40,41]. Variations in local microglial densities have also been identified in the human CNS parenchyma, with lower densities in the cerebellum compared to the mesencephalon and medulla oblongata, and higher densities in the white compared to grey matter [42,43]. Evidently, while a low expression of the F4/80 macrophage marker was found in the cerebellum, a similar expression of F4/80 was observed in the remaining brain regions [44], suggesting that microglia localised in the cerebellum may have a distinct role compared to other microglia populations [45].

The heterogeneity of microglia in the CNS is largely shaped by local cues resulting from the interaction with neighbouring neuronal and astrocytic cells [46]. For example, the depletion of interleukin (IL)-34, a colony-stimulating factor 1 receptor (CSF1R) ligand mainly secreted by neurons, led to a decrease in microglial densities in the cortex and striatum, but not in the cerebellum or brainstem of mice [47,48]. Similarly, a reduction in IL-34 expression levels was observed in the inferior temporal gyrus but not in the cerebellum of AD post-mortem brain tissues [49]. How microglia was implicated in the affected brain region with reduced IL-34 was not investigated in this study. However, since IL-34 regulates microglial proliferation, survival and the inflammatory response [49], it is likely that alterations in IL-34 in the brain may influence microglial activation, contributing to disease progression and pathogenesis in specific brain regions [50]. 

Similarly, the astrocyte-derived IL-33 was shown to modulate microglial synaptic engulfment specifically in the thalamus and spinal cord of a developing mouse brain [51]. Differences between particular microglia subsets, such as those located in the brain and the spinal cord, could result from the exposure to extrinsic blood-borne molecules that penetrate the blood-brain barrier [52]. Additionally, epigenetic mechanisms have been shown to tightly regulate microglial characteristics in a region-specific manner. A study performed in the adult mouse brain has shown that the phagocytic activity of striatal and cortical microglia is epigenetically supressed by the polycomb repressive complex 2 (PRC2) as opposed to cerebellar microglia [53]. 

Importantly, this regional microglial diversity could contribute to the onset, development and treatment response of neurological diseases, with a specific spatial pattern, such as PD [41]. Indeed, mouse microglia from the ventral tegmental area and substantia nigra pars compacta showed low cell numbers, sparse branching, reduced lysosome abundance and depressed cell metabolism [41]. These characteristics limit the capacity of microglia to support tissue homeostasis in this region, thereby contributing to an increased neuronal susceptibility and potentially leading to differential responses towards pathological triggers [54].

### 4.3. Microglial States Associated with Ageing

Over the course of CNS development under steady-state conditions (spanning from embryonic to ageing stages), microglia experience a dynamic change in phenotype. Early during development, microglia reduce their immune sentinel function and promote their phagocytic and synaptic pruning abilities for the formation of a mature CNS architecture [55]. In aged brains, microglia exhibit a higher sensitivity to immune stimuli, as well as enhanced inflammatory and interferon-responsive profiles [56]. This phenomenon has been described as microglia priming [57]. Similarly, aged microglia are implicated in the development of age-related neurodegenerative diseases [58]. Such increased immune reactivity may be due to cellular senescence, which alters the intrinsic mechanisms controlling the main microglial functions [59]. Indeed, during ageing, transforming growth factor (TGF)-β signalling decreases in microglia and enhances the responsiveness to ageing cues [60]. This impairment of microglial TGF-β signalling has been associated with a decrease in protective microglial functions and a potentiation of microglia-mediated neurodegeneration [60].

Aged microglia exhibit distinctive phenotypic alterations, including the upregulation of pathways associated with DNA damage, telomere maintenance and phagocytosis [61]. At the morphological level, microglia from post-mortem elderly individuals showed a loss of ramifications, the appearance of cytoplasmic fragmentation and shortening of cellular processes [62] without alterations in cell density [63]. At the functional level, aged microglia showed a decrease in chemotaxis, process motility and migration towards extracellular ATP [64]. A compromised capacity to clear myelin fragments has also been reported in microglia from aged mice. Indeed, myelin engulfment by microglia led to the build-up of insoluble, lipofuscin-like lysosomal inclusions that triggered immune dysfunctions and cell senescence [65]. In line with this finding, a recent study described an age-dependent accumulation of lipid droplets in microglia from aged mouse and human brains [66]. Such microglia, termed ‘lipid-droplet-accumulating microglia’ (LDAM), were found to express a unique transcriptomic signature indicative of deficient phagocytosis and an enhanced pro-inflammatory phenotype [66]. Like LDAM, other age-associated microglial states with a unique transcriptomic profile have been described. These include ‘activated response microglia’ (ARM), which made up the 12% of total microglia in aged mice and showed an upregulation of histocompatibility complex class II and pro-inflammatory genes [67]. In the same study, a concomitant microglia subset, termed ‘interferon-response microglia’ (IRM), was identified. IRM displayed an upregulation of interferon-response genes [67]. Importantly, age-related changes in microglia can lead to dysregulated microglial states that trigger circuitry dysfunction. This promotes the development of neurodegenerative diseases and neuropathological conditions such as age-associated cognitive decline [56,57].

Microglia are especially susceptible to the ageing process because of their longevity and slow turnover rate throughout adult life [39,68]. During ageing, the CNS microenvironment becomes more hostile, with increased tissue injury, cell death and foreign stimuli. Further, as the BBB permeability increases with age, peripheral insults resulting from infectious agents or chronic metabolic diseases become more prevalent in the CNS [69,70]. Collectively, these factors drastically change the chemical composition of the CNS parenchyma [71], releasing cues that may potentially drive the genetic and functional alterations observed in aged microglia. 

### 4.4. Microglial States Associated with Sex

Interestingly, sex has been described to modulate microglial features at various levels, including cell density, size and phagocytic function [72,73]. Significant sex-specific differences in the number and morphology of microglia were observed in various regions of the mouse brain, such as the preoptic area, hippocampus, parietal cortex and amygdala [72,74]. For example, male hippocampal microglia exhibited a more complex morphology, including an increased number, volume and area of cell processes, than their female counterparts [75]. Microglial gene expression profiles and function also exhibit striking differences between males and females during development and ageing [75,76,77].

Sex-related differences in microglia could explain the sexual dimorphism observed in diseases such as AD. In a recent study, microglia from female transgenic AD mice showed a more profound metabolic shift towards glycolysis and an enhanced deterioration of phagocytic activity than compared to male AD mice [78]. When assessing post-mortem brain tissue samples, AD patient microglia exhibited diverse morphologies, including a few amoeboid cells, some ramified cells and numerous rod-shaped cells in female patients [78]. In contrast, microglia from male AD patients were predominantly amoeboid and showed increased CD68 immunoreactivity, suggesting a greater phagocytic function than female microglia [78]. The authors proposed that the observed sex-related differences in microglia could underlie the marked amyloid pathology, reduced neuroprotection and greater cognitive impairment in female AD patients. 

### 4.5. Microglial States Associated with Neurological Disease

During neurological disease, microglia organise into context-dependent heterogeneous subpopulations that react to surrounding and specific pathological alterations. For example in the brains of AD transgenic mouse models, microglia clustered around extracellular Aβ plaque deposits, called disease-associated microglia (DAM), showed a unique genetic and functional signature. This DAM signature involves the upregulation of lysosomal, phagocytic and lipid metabolism pathways and is also observed in microglia from ALS mouse models [79,80]. Interestingly, data from single-nuclei RNA sequencing (snRNAseq) revealed the presence of specific microglia states within post-mortem human AD brains not seen in mouse AD models. Specifically, a subpopulation of microglia termed Mic1 expressed the AD-associated genes *C1QB* and *CD14* as a unique feature of human compared to animal disease models [81]. The Mic1 population was also significantly enriched in female compared to male AD patient brains [81]. Another study, which reported the transcriptomic profiles of three female AD-patient post-mortem brains using snRNAseq, identified the expression of only 92 genes out of the 500 DAM-associated genes previously detected in mouse microglia [82]. Accordingly, the transcriptomic profiling of frozen human cortical brain tissue from ten AD patients indicated few commonalities between the human and mouse AD microglial gene signatures [83]. The authors named this newly identified microglia subset human Alzheimer’s microglia (HAM). While HAM only shared the high expression of lipid/lysosomal biology-related genes such as *APOE*, *LSR* and *ARSA* with DAM, a striking overlap was observed with microglia from aged individuals [83]. This signature included the high expression of genes such as IL15, *MS4A6A*, *MS4A4A*, *NME8*, and *GPR141*. The authors concluded that HAM appear in AD patient brains as a result of enhanced ageing and age-independent, disease-related activation processes [83].

In other neurodegenerative diseases such as PD, microglia from patients showed substantial heterogeneity specific to brain regions. For example, microglia located in the substantia nigra, the most pathologically impacted region in PD, exhibited different transcriptomic profiles compared to microglia located in the prefrontal cortex [84]. The altered biological processes observed in PD patient microglia involved behavioural responses to stimuli, the regulation of transport and synaptic transmission [84]. In ALS brain samples, both derived from mouse models and patients, a selective increase in keratan sulphate proteoglycan (KSPG)-microglia was described [85,86]. KSPG-microglia are believed to control cellular adhesion and axonal growth and could be protective during the early pathogenesis of ALS [87,88].

Importantly, the microglial states identified in neurodegenerative diseases are different from models of inflammation. For example, a global downregulation of microglial homeostatic genes with an upregulation of inflammatory genes was observed in a mouse model of systemic inflammation, [89]. In contrast, only a subset of microglia was highly proliferative and displayed an enhanced capacity for leukocyte recruitment in experimental autoimmune encephalomyelitis (EAE), a model of demyelinating diseases [56,90].

Overall, it is evident that disease-related perturbations can cause dramatic changes in microglial phenotypes, giving rise to heterogeneous microglia subsets that show greater diversity than in steady-state CNS conditions.

## 5. Limitations of Primary and Immortalised Microglia In Vitro Models to Study Neurodegenerative Disease

Modelling the identity of human microglia while accounting for specific signatures derived from the brain region, sex, ageing and disease states is a major challenge. In fact, current microglia model systems are limited in terms of capturing these particular microglial characteristics. Microglia are commonly studied using in vivo models derived from rodents [91] and in vitro models of primary and immortalised rodent and human microglia cell lines [92] (Table 1).

The use of primary and immortalised in vitro models has greatly expanded our understanding of microglial biology; however, the successful translation from disease models to human clinical trials is far from adequate [93]. Aspects that hamper the translatability of these models into patients include (1) human-specific neurodegenerative disease traits, which limit the validity of rodent models, and (2) the reduced availability and quality of human cell samples, which introduces biases in research outcomes obtained by using primary and immortalised human microglia cell models. The advent of stem cell-derived approaches has been paramount in overcoming the shortcomings of primary and immortalised microglia in vitro models. Compared to primary and immortalised lines, stem cell-derived microglia-like cells recapitulate authentic human microglial features and allow for the maturation of the cells in the presence of CNS-derived cues (Table 1).

The following subsections describe the two main limitations faced by rodent microglia model systems and human microglial cells (primary and immortalised) when used to study neurodegenerative diseases.

**Table 1:** Characteristics of primary, immortalised and stem cell-derived microglia cell model systems.

		Donor Characteristics	Source	Culture Conditions	Phenotypic Characteristics	Advantages/Disadvantages	Applications	Studies
**Primary microglia**								
***Human***		- Foetal/Aged adults - Neurological disease (healthy donors limited)	Tissue source: autopsy, biopsy Autopsy tissue conditions: - ante-mortem: uncontrollable - post-mortem: delay of 2–20 h Yield: 200,000–500,000 cells/gram of tissue	10% FBS in DMEM/F12	Freshly isolated cells: - Low and absent expression of *CD45* and *CCR2*, respectively - High expression of microglia-enriched genes (*P2RY12, TMEM119, TREM2, GPR34, CX3CR1, C1QA, PROS1, GAS6*) Cultured cells: - Downregulation of mature microglia markers (*P2RY12, TMEM119*) - Amoeboid morphology - Inflammatory activation (enhanced secretion of inflammatory cytokines and nitric oxide) - Highly proliferative	Advantages: - Patient-specific - Best correlate to in vivo microglia when freshly isolated Disadvantages: - Tedious and time-consuming isolation procedure - Limited resource - Ethically challenging to obtain - Low sample yields (cells, RNA) - Low purity cultures (i.e., 5% of astrocytes and oligodendrocytes) - Variable activation states depending on isolation method	Freshly isolated cells: - Transcriptomic and proteomic profiling of microglia for investigating disease-related phenotypes - Characterise microglial heterogeneity associated with brain region and sex Culturedcells: Study of microglia physiology in vitro (e.g., surveillance, phagocytosis, immune activation)	[83,94,95,96]
***Rodent***		- Neonatal/Adult animals - Wild type and transgenic animals	Tissue conditions: - ante-mortem: controllable - post-mortem: no delay Yield: 500,000-700,000 cells/gram of tissue	10% FBS in DMEM/F12		Advantages: - Controlled genotype Disadvantages: - Interspecies differences on genes related to immune function and ageing (e.g., *SIGLECs, MHCs, TLR4, IFN-γ, IL-15, CD33*), limiting translation to humans - Limited yields, impeding high-throughput assays - If cultured long-term, potential for proliferation of contaminating cells (e.g., pericytes)		[79,97]
**Immortalised microglia cell lines**								
***Human***	HMO6	Embryonic Transformed, *v-myc* oncogene		10% FBS in DMEM/F12	Attenuated or lack of response to inflammatory stimuli (e.g., neither release of IL-1β nor nitric oxide)	Advantages: - Easy to maintain - Unlimited availability - Homogenous microglia populations Disadvantages: - Genetically altered - Prone to dedifferentiate - Less sensitive to inflammatory stimuli than primary microglia - Subject to genetic drift and morphology changes	- Biochemical and molecular studies - Pharmacological studies - High-throughput screening assays	[98]
	HµGlia	Adult Transformed, SV40 large T antigen and hTERT		10% FBS in DMEM/F12	Lack expression of microglia-enriched genes			[99]
	CHME-5	Embryonic Transformed, SV40 large T antigen		10% FBS in DMEM/F12	Uncertain origin (rat origin suggested)			[100]
	HMC3	Derived from CHME-5 line		10% FBS in EMEM	Lack expression of microglia-enriched genes			[101]
	C13NJ			10% FBS in DMEM/F12				[102]
	SV40 (IM-HM)	Embryonic Transformed, SV40 large T antigen		20% FBS	Low expression of microglia-enriched genes			[103]
***Mouse***	BV2	Neonatal Transformed, *v-raf/v-myc* oncogene		10% FBS in DMEM	Attenuated response to inflammatory stimuli (e.g., no release of IL-1β)			[104]
	N9, N11	Embryonic Transformed, *v-myc* oncogene		10% FBS in DMEM	Express a limited number of inflammatory mediators			[105]
	EOC (subtypes 2, 13.31, 20)	Neonatal Spontaneously immortalised		10% FBS in DMEM with M-CSF supplement	Some subtypes do not express MHCII			[106]
	IMG	Adult Transformed, *v-raf/v-myc* oncogene		10% FBS in DMEM	Amoeboid morphology			[107]
***Rat***	HAPI	Neonatal Spontaneously immortalised		10% FBS in DMEM	Attenuated response to inflammatory stimuli			[108]
**Stem cell-derived microglia**								
***Human***	- Healthy adults - Neurological disease	**hiPSCs** (derived from genetically reprogrammed somatic cells, such as skin fibroblasts)		Differentiation towards microglial lineage has been achieved in: - *2Dmono-culture* - *2D/3Dco-culture with neurons* - *Xenograft in mouse brain(humanised in vivo model)* Culture medium is commonly supplemented with M-CSF, IL-34, SCF, VEGF, BMP4, ActivinA and TPO	Best resemble foetal or early postnatal microglia when differentiated under 2D mono-culture conditions (i.e., low expression of *TREM2*, *TMEM119* and *P2RY12* compared to adult microglia)	Advantages: - More authentic microglia phenotype than immortalised cell lines - Unlimited availability of patient material - The hiPSC-derived model allows for the generation of isogenic controls - Possibility to study human microglia in vivo with humanised mouse models - In co-culture models: assess maturational effects derived from the contact with other brain cell types Disadvantages: - Long differentiation times - Differentiation method might not faithfully recapitulate the MYB-independent ontogeny of microglia - In mono-culture models: lack of physiologically relevant cell–cell and cell-extracellular matrix interactions	- Characterisation of patient-specific disease states in microglia - Investigation of the interaction between microglia and other brain cell types - Stratification of patient drug responses - Development of personalised medicine approaches	[109,110,111,112,113]
		**Monocytes** (isolated from peripheral blood)		RPMI with GM-CSF and IL-34 supplements (Elaborated in Table 2)				[114,115,116,117,118]

hTERT: human telomerase gene; MHCII: major histocompatibility complex class II; hiPSCs: human-induced pluripotent stem cells; FBS: foetal bovine serum; 2D: two dimensional; 3D: three dimensional.

## 5.1. Interspecies Differences of Microglia Neurodegenerative Disease Phenotypes

Microglia exhibit strong species-specific features under disease and ageing conditions [126]. Specifically in the context of age-related neurodegenerative diseases, discrepancies in microglia disease phenotypes between mouse models of AD and patients have been identified (*for an in-depth review see* [127]). The activation signature displayed by microglia from AD transgenic mouse models does not correlate with that of patients, where microglia exhibit transcriptional traits typical of a senescent, rather than an activated, phenotype [83]. Human AD microglia also upregulate responses against viral and bacterial infections that are absent in microglia from AD mouse models [126]. Similarly, microglial responses to Aβ pathology are different between disease models and patients, with AD mouse microglia exhibiting a robust DAM signature and AD patient microglia adopting an interferon regulatory factor 8 (IRF8)-driven gene signature [35]. Indeed, a limited overlap between the DAM gene profile identified in AD mouse models and the transcriptomic signature of AD patient microglia has been consistently reported [81,82,83]. Lastly, some AD risk genes expressed by human microglia, such as *CD33*, *MS4A* and *CR1*, lack mouse orthologues [128], supporting the limitations of mouse models as tools to investigate AD pathways relevant to the human disease.

The reasons for the disparities between microglia from AD mouse models and patients are unclear. It is possible that mouse AD microglia reflect changes from early disease stages that are not present in the human brain specimens used for comparison. In addition, the disease is modelled in mice by inducing an artificial overexpression of pathological proteins within a short timeframe that poorly reflects the slow and cumulative course of pathological events occurring in humans. This could make microglial responses between mouse models and humans not comparable, hindering the progress of research in the field of neurodegenerative diseases, and limiting the design of efficient therapeutic approaches.

## 5.2. Limited Availability and Quality of Primary and Immortalised Human Microglia

One of the major limitations of transitioning microglia studies from mice to cultured primary human cells is the scarce availability of human brain tissue. Because available tissues are obtained at autopsy or during neurosurgical procedures, it is difficult to gather a large enough number of samples from patients with similar disease states. Another important limitation is that microglial purification procedures from resected brain tissue require a multistep methodology that rapidly alters cell properties. As culture conditions hardly mirror the brain environment, the altered phenotype in isolated microglia is not restored to an in vivo state [29]. Immortalised human microglia cell lines offer an alternative to primary cultures, providing a limitless source of cells. However, they do not recapitulate disease traits, easily dedifferentiate, and lack physiological relevance due to viral transduction [129].

## 6. Improving Current Microglia Cell Models

Despite the limitations in using primary and immortalised microglia, these microglia models have provided a platform to answer various fundamental questions in microglial biology in health and disease. With advanced tools in the field, new human-relevant cell culture approaches, such as patient human-induced pluripotent stem cells (hiPSC)-derived models and patient monocyte-derived microglia (Table 1), can now aid in generating a more relevant and comprehensive picture of microglial behaviours in the context of neurodegenerative disease. By circumventing the drawbacks of isolation and immortalisation procedures, patient stem cell-derived microglia model systems may be a better tool for a successful clinical translation.

### 6.1. Generating Microglia from Patient-Derived hiPSCs

Stem cell technology has enabled the generation of microglia containing the patient’s specific genetic background by using a renewable pool of pluripotent cells. These cells, known as hiPSCs, can be efficiently directed towards microglia-like phenotypes [110,111,130,131,132]; *for review see* [133,134]). hiPSC-derived microglia protocols mimic the embryonic developmental lineage of microglia by generating hematopoietic progenitors and exposing them to key signals. The resulting microglia-like cells assume immature characteristics resembling foetal or early post-natal human microglia. Although several protocols exist to generate hiPSC-derived microglia, there is no consensus on which method is the most effective and reproducible. Additionally, most methods involve lengthy procedures, where the shortest is 24 days in culture [135], and hiPSCs accumulate genetic instability upon increased passaging and time in culture [136]. Another caveat of hiPSCs-derived cellular models is that studies are limited to small patient cohorts, as the cost of hiPSC derivation and differentiation is high. Lastly, hiPSC reprogramming may cause the loss of disease-relevant epigenetic traits [137].

### 6.2. Generating Microglia from Patient-Derived Monocytes

An alternative method to generate patient-specific microglia-like cells is the direct transdifferentiation of monocytes isolated from peripheral blood. All monocyte-derived microglia methods commonly use CSF1R ligands to promote the induction, proliferation, and survival of microglial cells [103,114,115,116,117,118,120,121,123,124,125,138] (Table 2). In contrast to hiPSC-derived microglia protocols, monocyte-derived microglia techniques circumvent genetic engineering (needed to generate hiPSCs from somatic cells), require short culture periods (maximum 14 days) and allow for longitudinal studies of large patient cohorts. Similar to hiPSC-derived microglia, monocyte-derived microglia exhibit foetal properties [103].

Some have argued that hiPSC-derived microglia-like cells more closely resemble brain-resident microglia, as they better mimic the ontogeny of embryonic microglia compared to monocyte-derived microglia [134]. Ontogenically, microglia originate from yolk sac-derived erythromyeloid progenitors in the embryo, whereas monocytes derive from bone marrow hematopoietic precursors in the adult. However, during neurodegenerative disease, monocytes can infiltrate the brain parenchyma, possibly in a chemokine receptor (CCR)-2-dependent manner, and differentiate into microglia-like cells [139]. In AD, this pool of peripheral-derived microglia-like cells is recruited to sites of amyloidosis and clears protein deposits more effectively than resident microglia, which easily die in the presence of pathological stressors [140,141]. A recent study has demonstrated that peripheral-derived microglia-like cells exhibit unique transcriptional and functional features compared to embryonic microglia [142]. Importantly, these peripheral-derived microglia-like cells may be capable of replacing microglia under deficiency conditions, such as neurodegenerative disease brains, where a portion of resident microglia are likely senescent [62]. Nevertheless, the monocyte-derived microglia model represents a valuable in vitro model to study microglia in the context of neurodegenerative diseases.

### 6.3. How Do the Microglia-like Cell Characteristics of hiPSC- and Monocyte-Derived Microglia Models Compare to Each Other?

Both hiPSC- and monocyte-derived microglia exhibit microglia-like features, including the expression of signature markers, phagocytic capacity, and the ability to mount an inflammatory response. However, whether the different cell origin and differentiation methodologies between the models affect microglia-like cell phenotypes at other levels has been scarcely investigated. A recent study directly compared monocyte-derived microglia to hiPSC-derived microglia using RNA sequencing and found that the transcriptomic signatures of both models clustered together [123,130,143]. Interestingly, differences among hiPSC-derived models were noted, highlighting the challenges of identifying optimal differentiation procedures using hiPSCs [143]. A study modelling human immunodeficiency virus (HIV) infection in microglia compared monocyte-derived microglia to hiPSC-derived microglia and identified functional differences in viral replication and particle internalisation between the models [144]. This suggests that, despite showing similar transcriptomic profiles, hiPSC- and monocyte-derived microglia-like cell models respond differently to immune challenges. Whether both models exhibit divergent behaviours in a neurodegenerative disease background deserves further investigation.

The use of both hiPSC- and monocyte-derived microglia-like cell models has yielded valuable discoveries on human microglial physiology in homeostasis and disease. This shows that both cell models are useful to further our understanding of the involvement of microglia in disease. Both microglia-like cell models have shown applicability for a diverse range of studies. For example, hiPSC-derived microglia-like cells have been used to dissect signalling pathways linked to mutations in disease-relevant receptors and transporters, including TREM2, APOE and NPC1 [135,145,146]. Moreover, the use of hiPSCs offers the possibility of generating different cell types from the same donor. On the other hand, monocyte-derived microglia have been used to study patient microglial phenotypes from various diseases, such as Nasu–Hakola, schizophrenia, fibromyalgia, ALS and Huntington’s disease [103,114,119,125,147]. Compared to hiPSC-derived models, monocyte-derived microglia offer the flexibility to model large patient cohorts and hence are better suited for genetic and drug screening studies [115]. In summary, both cell models hold promise to progress microglia research.

## 7. Applications of Patient-Derived Microglia In Vitro Models to Study Neurodegenerative Diseases

Patient-specific microglia models are promising translational research tools for the study of microglia-relevant disease mechanisms that can serve as readouts for drug screening. Some of the altered microglial phenotypes identified in patient hiPSC- and monocyte-derived microglia-like cell model systems include aberrant synaptic pruning, exacerbated inflammatory responses, and deficient migratory and metabolic activities (Table 3). The spectrum of modelled diseases is broad, ranging from autoinflammatory disorders and SARS-CoV-2 infection to neurological conditions including schizophrenia and dementia.

In addition to their application as in vitro platforms for the discovery of druggable disease phenotypes, patient-derived cellular microglia models may potentially bridge the gap between clinical studies. Firstly, molecular data from in vitro cultures of one patient (e.g., cell morphology, phagocytic activity, inflammatory cytokine secretion, and responsiveness to stressors) can be correlated with the clinical data of that same patient (i.e., brain imaging and clinical disease progression). Such a correlation can help inform drug efficacy studies in discriminating between drug responders and non-responders and stratify patients in clinical studies [153]. Secondly, biomarkers of disease progression can be monitored by longitudinally investigating cultures that have been generated at various disease stages. Lastly, the impact of genetic risk variants can be assessed in cases of mild or early disease progression.

## 8. Conclusions

Increasing evidence has demonstrated the implications of microglial activation in driving the onset and progression of neurodegenerative diseases. Yet, the overall success of microglia-targeted therapeutics for neurodegenerative diseases remains very low. The reasons for this failure include the lack of an accurate microglia model system able to reflect: (1) patients’ heterogeneity at the clinical and genetic levels; (2) clinical heterogeneity over the course of disease; (3) key pathological hallmarks; (4) inter-species differences in microglial homeostatic and activation expression profiles; and (5) differences between microglia models (isolated from post-mortem brain, immortalised cell lines, patient-derived hiPSCs, and patient-derived monocytes). It is imperative to recognise the strengths and limitations of current model systems to further expand our knowledge on the role of microglia as well as to improve and strengthen translational outcomes in neurodegenerative diseases.

Indeed, the use of microglia cells alone is insufficient for a successful translation from bench-to-bedside. Current patient-derived microglia cultures as well as other in vitro model systems are established in a 2D environment that poorly resembles their in vivo counterparts [154]. An enhanced microglial platform established using 3D modelling that incorporates a brain microenvironment with various neuronal cell types is required to better mimic the complexity of the brain homeostatic and diseased states, which has been exemplified in several studies [130,138,155]. This in turn would provide better pre-clinical predictions and outcomes and thereby increase translational potential.

## Figures and Tables

**Table 2:** Published methods to generate monocyte-derived microglia-like cells.

	Leone 2006 [116]	Etemad 2012 [118]	Ohgidani 2014, 2017 [114,119]	Melief 2016 [117]	Ryan 2017 [115]	Sellgren 2017 [103]	Rawat 2017 [120]	Sellgren 2019 [121]	Ormel 2020, [122]	Banerjee 2021 [123]	Smit 2022 [124]	Quek 2022 [125]
**Supplements**	Astrocyte conditioned medium: 25% GM-CSF: 1 ng/mL M-CSF: 10 ng/mL	Astrocyte conditioned medium: 25% M-CSF: 10 ng/mL NGF-β: 10 ng/mL CCL2: 100 ng/mL	GM-CSF: 10 ng/mL IL-34: 100 ng/mL	GM-CSF: 10 ng/mL IL-34: 100 ng/mL	M-CSF: 10 ng/mL GM-CSF: 10 ng/mL NGF-β: 10 ng/mL CCL2: 100 ng/mL IL-34: 100 ng/mL	GM-CSF: 10 ng/mL IL-34: 100 ng/mL	M-CSF: 10 ng/mL NGF-β: 10 ng/mL CCL2: 100 ng/mL	GM-CSF: 10 ng/mL IL-34: 100 ng/mL	Astrocyte conditioned medium: 25% GM-CSF: 10 ng/mL M-CSF: 10 ng/mL TGF-β: 20 ng/mL IL-34: 10 ng/mL IFN-γ: 12.5 ng/mL	GM-CSF: 10 ng/mL IL-34: 100 ng/mL	Astrocyte conditioned medium: 25% GM-CSF: 10 ng/mL M-CSF: 10 ng/mL TGF-β: 1 ng/mL IL-34: 100 ng/mL IFN-γ: 12.5 ng/mL	GM-CSF: 10 ng/mL IL-34: 100 ng/mL
**Days**	12	14	14	14	15	11	10	11	10	10–14	10	14
**Seeding density**	T75 flask	1 × 10^5^ cells/mL	4 × 10^5^ cells/mL	4 × 10^5^ cells/mL	3 × 10^5^ cells/well (24-well plate)	500,000 cells/well (24-well plate)	50,000 cells/well (96-well plate)	1 × 10^6^ cells/well (24-well plate)	1 × 10^6^ cells/well (48-well plate)	1 × 10^6^/mL	600,000 cells/well (48-well plate)	500,000 cells/ well (48-well plate)
**Coating**	*N/A*	*N/A*	*N/A*	*N/A*	*N/A*	Geltrex	*N/A*	Geltrex	Poly-L-lysine	Geltrex	Poly-L-lysine	Matrigel
**Monocyte** **isolation**	Counterflow centrifugal elutriation	Adherence to plastic	Adherence to plastic	Anti-CD14+ microbeads	Anti-CD14+ microbeads	Adherence to plastic	Anti-CD14+ microbeads	Adherence to plastic	Anti-CD14+ microbeads	Adherence to plastic	Anti-CD14+ microbeads	Adherence to plastic
**Transcriptomic** **profiling**	No	No	No	No	RNAseq	Nanostring	No	Global gene expression by microarray	RNAseq	RNAseq	RNAseq	No
**Disease** **modelled**	*N/A*	*N/A*	Nasu–Hakola disease (2014) Fibromyalgia (2017)	*N/A*	*N/A*	Schizophrenia	HIV infection	Schizophrenia	Schizophrenia	*N/A*	*N/A*	ALS

*N/A* indicates information “not specified”.

**Table 3:** Studies that have modelled microglia disease phenotypes using patient-derived microglia in vitro models.

Disease	Microglia Model System	Number of Patients	Disease-Specific Characteristics Compared to Controls	Reference
FTD	**hiPSC-derived** **microglia**	1 patient with sporadic FTD 1 patient with familial FTD (progranulin gene mutation—PGRN S116X)	Reduced levels of intracellular and secreted progranulin	[148]
FTD-like syndrome Nasu–Hakola disease		1 patient homozygous for the TREM2 T66M mutation (FTD-like syndrome) 1 patient homozygous for the TREM2 W50C mutation (Nasu–Hakola disease)	Accumulation of immature TREM2 protein Absence of TREM2 protein on the plasma membrane Similar release of pro-inflammatory cytokines following LPS stimulation Similar phagocytic capacity	[149]
Nasu–Hakola disease		2 patients homozygous for the TREM2 T66M and W50C mutations	Reduced survival Similar release of pro-inflammatory cytokines following LPS stimulation Reduced phagocytosis of apoptotic neuronal cells Reduced release of cytokines mediating chemotaxis and chemoattraction Reduced migration towards apoptotic cells	[150]
AD (sporadic)		>1 * patient heterozygous for the TREM2 RH47H mutation	Reduced mitochondrial respiratory capacity Reduced phagocytosis of Aβ Inability to perform a glycolytic immunometabolic switch	[146]
			Reduced activation of NLRP3 inflammasome	[151]
Familial Mediterranean fever		1 patient	Increased release of pro-inflammatory cytokines following LPS stimulation Upregulated ASC-speck formation	[112]
AD (familial and sporadic)		2 patients with the APP KM670/671NL Swedish mutation (familial AD) 2 patients with the PSEN1 PSEN1ΔE9 mutation (familial AD) 4 patients with APOEƐ4/4 (sporadic AD)	Familial lines: - Reduced release of pro-inflammatory cytokines following LPS stimulation - Similar mitochondrial metabolism - Increased chemokinesis Sporadic lines: - Increased release of pro-inflammatory cytokines following LPS stimulation - Reduced mitochondrial metabolism - Reduced chemokinesis	[135]
**Nasu–Hakola** **disease**	**Monocyte-derived** **microglia**	1 patient	Delayed release of pro-inflammatory cytokines following stimulation with latex beads	[114]
**Schizophrenia**		13 patients	Increased phagocytosis of synaptic material	[121]
**Fibromyalgia**		14 patients	Increased mRNA and protein expression of TNF-α following ATP stimulation	[119]
**Schizophrenia**		20 patients	Increased release of pro-inflammatory cytokines following LPS stimulation	[122]
**Huntington’s** **disease**		6 pre-manifest gene carriers 6 manifest gene carriers	No differences compared to microglia-like cells from matched healthy controls	[147]
**SARS-CoV-2** **infection**		2 patients (neonates exposed to maternal SARS-CoV-2 infection during pregnancy)	Phagocytosis of synaptic material No direct comparison with microglia-like cells generated from neonates from healthy mothers	[152]
**ALS** **(sporadic)**		30 patients (including patients with slow, intermediate and rapid disease progression)	Impaired phagocytosis that correlates with disease progression Altered cytokine profiles Altered morphology Increased DNA damage and NLRP3 inflammasome activity	[125]

* The exact number of patient cell lines is not specified. FTD: frontotemporal dementia; NLRP3: NOD-leucine rich repeat and pyrin containing protein 3.

## Data Availability

Not applicable.
